# Multiwavelength Photoacoustic Breath Analysis Sensor for the Diagnosis of Lung Diseases: COPD and Asthma

**DOI:** 10.1021/acssensors.3c01316

**Published:** 2023-10-23

**Authors:** Nidheesh V. R., Aswini Kumar Mohapatra, Vasudevan Baskaran Kartha, Santhosh Chidangil

**Affiliations:** † Centre of Excellence for Biophotonics, Department of Atomic and Molecular Physics, Manipal Academy of Higher Education, Manipal 576104, Karnataka, India; ‡ Department of Respiratory Medicine, Kasturba Medical College, Manipal, Manipal Academy of Higher Education, Manipal 576104, Karnataka, India

**Keywords:** multiwavelength PAS spectroscopy, volatile organic compounds, breath analysis, multivariate analysis, diagnosis

## Abstract

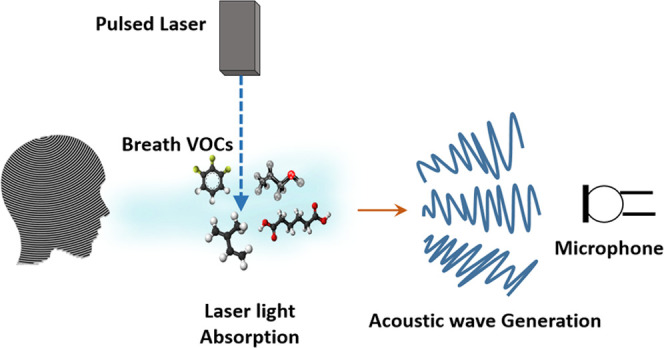

Breath analysis is emerging as a universal diagnostic method for clinical applications. The possibility of breath analysis is being explored vigorously using different analytical techniques. We have designed and assembled a multiwavelength UV photoacoustic spectroscopy (PAS) sensor for the said application. To optimize laser wavelength for sample excitation, photoacoustic signals from disease and normal conditions are recorded with different laser excitations (213, 266, 355, and 532 nm) on exhaled breath samples. Principal component analysis (PCA) of the PA signals has shown that 213, 266, and 355 nm laser excitations are suitable for breath analysis, with reliable descriptive statistics obtained for 266 nm laser. The study has, therefore, been extended for breath samples collected from asthma, chronic obstructive pulmonary disease (COPD), and normal subjects, using 266 nm laser excitation. PCA of the PA data shows good classification among asthma, COPD, and normal subjects. Match/No-match study performed with asthma, COPD, and normal calibration set has demonstrated the potential of using this method for diagnostic application. Sensitivity and specificity are observed as 88 and 89%, respectively. The area under the curve of the ROC curve is found to be 0.948, which justifies the diagnostic capability of the device for lung diseases. The same samples were studied using a commercial E-Nose, and the measurement outcome strongly supports the PAS results.

Breath analysis is getting much attention for research and development as an alternative diagnostic tool for many diseases due to its noninvasive nature and relatively fast, cheaper technology. The volatile organic compounds (VOCs), emanating from different body parts due to various metabolic activities, finally reach the lungs through blood circulation and are present in exhaled breath. Exhaled breath from healthy volunteers and subjects with a disease condition can thus be analyzed by this method to find diagnostic VOC markers for the disease..^1−5^ Analytical devices such as gas chromatography–mass spectrometry (GC-MS), millimeter-wave gas spectroscopy, laser spectroscopy, and sensors like E-nose have been explored for breath analysis.^6−10^ Among the laser spectroscopy techniques, photoacoustic spectroscopy (PAS) is a sensitive and promising tool for breath analysis.

The excitation of the molecule on the absorption of the laser radiation in the photoacoustic cell can result in its elevation to higher electronic/vibrational/rotational states. In other words, acoustic waves can be generated by the absorption of molecular species in distinct regions: UV–Vis (ultraviolet–visible) (due to electronic transitions), IR (infrared) (due to vibrational transitions), or Microwave (pertaining to rotational transitions). The acoustic signal is directly linked to the molecules’ absorption and can be detected using sensitive microphones.^11^ Often, the technique’s detection limits can extend into the range of parts per million (ppm) to parts per billion (ppb) or even lower.

The increased accessibility of adjustable quantum cascade lasers within the infrared spectrum has greatly expanded the potential applications of PAS.^12^ IR-based PAS sensors have been explored to study renal failure, cystic fibrosis, cardiac conditions, etc.^13−16^ High scan speed and precise tunability (with a small range) are the advantages of IR-based systems. The absorption cross sections of the molecules exhibit substantial variation in the chosen region of IR radiation. Despite the fact that numerous molecules exhibit robust absorption in the mid-IR region, there are numerous advantages associated with UV excitation for PAS investigations. In the UV range, the energy relaxation contributing to the PAS signal is significantly greater per absorbed photon. For instance, numerous VOCs observed in exhaled breath demonstrate pronounced absorption in the 200–300 nm range.^17^

Conversely, major components found in exhaled breath, such as water, N_2_, O_2_, and CO_2_ do not absorb in this range. In contrast, the mid-IR region has strong absorption by CO_2_ and water, which can introduce substantial interference into PAS studies of VOCs. In such cases, presample treatments would be necessary, making the technique more complex. Also, UV-PAS spectroscopy has opened new avenues for miniaturization by utilizing cost-effective LEDs for point-of-care (PoC) applications.

Exhaled breath contains a wide array of VOCs, encompassing numerous aldehydes, ketones, aromatic compounds, and other molecules, such as NO, CO, NH_3_, etc., which exhibit electronic absorption in the UV–B/C regions. Thus, exploring different wavelengths for electronic transitions and choosing a suitable single or combination of two or more wavelengths for this purpose will be helpful. In this work, therefore, we employed a pulsed laser with wavelengths of 213, 266, 355, and 532 nm for the PAS study of breath samples. A diagram illustrating the breath analysis approach employing the PAS configuration is shown in Figure 1.

**Figure 1:**
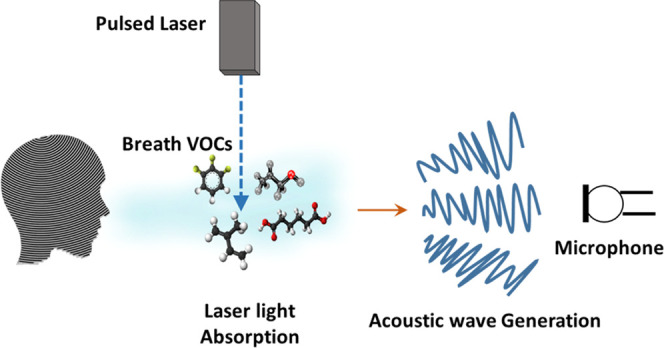
Schematic representation of breath analysis using the PA method.

Among lung diseases, asthma is a chronic condition that affects individuals of all ages. In this ailment, inflammation and the constriction of muscles around the small airways lead to the narrowing of the air passages in the lungs.^18^ This causes a cough, wheezing, shortness of breath, and chest tightness. Current diagnostic methods include X-ray, spirometry, and peak flow measurement. Breath analysis stands as a rapid and noninvasive diagnostic technique for asthma, as the asthmatic condition is anticipated to trigger the release of numerous marker VOCs from the lungs.^19^ Chronic obstructive pulmonary disease (COPD), is a persistent inflammatory lung condition characterized by restricted airflow in the lungs.^20^ Common symptoms of COPD encompass breathlessness, chest tightness, persistent cough, frequent respiratory infections, and a notable lack of energy.^21^ COPD condition may be contributed by emphysema and chronic bronchitis. Smoking tobacco and air pollution are the leading causes of COPD. COPD is a progressive disease, and diagnosis in earlier stages can help with better treatment. Current diagnosis methods include lung function tests (e.g., spirometry, lung volume capacity test, and pulse oximetry), chest X-ray, CT scan, arterial blood gas analysis, laboratory tests for checking α-1-antitrypsin deficiency (which may be the cause of COPD in some people), etc.

So far, the fractional exhaled NO (F_E_NO) test is a routine breath analysis method for diagnosis of COPD and asthma. In the F_E_NO test, the amount of nitric oxide in breath is measured, which can be used to confirm airway inflammation, which is a sign of lung infection. This method currently lacks information regarding other breath biomarkers. A device utilizing the PAS principle has the capacity to detect numerous VOCs in exhaled breath, potentially yielding more comprehensive results that can enhance both diagnosis and the classification of diseases.

In an earlier study, we described the design and development of a PAS sensor for breath analysis and preliminary results on analyzing exhaled breath in asthma samples.^17^ In most cases, both COPD and asthma have similar symptoms, and it is necessary to differentiate between them to provide appropriate therapy. This manuscript explores a multiwavelength study of breath samples to select a suitable source of excitation. Also, the assembled PAS sensor with 266 nm laser excitation is evaluated using clinically tested asthma, COPD, and normal samples. Multivariate data analysis is carried out with the PAS data. Experiments with the same samples were repeated using an E-nose to further confirm the results obtained from PAS.

## Materials and Methods

### Experimental Setup

**Figure 2:**
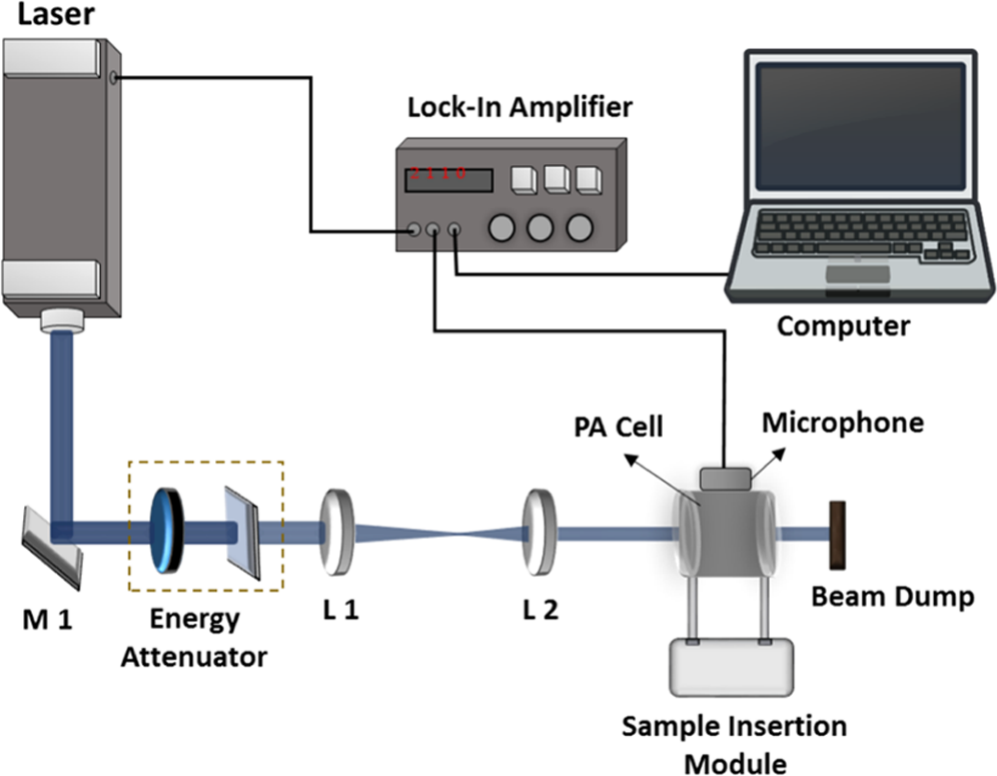
PAS experimental setup for breath analysis.

The PAS system consists of a pulsed laser as an excitation source and a microphone coupled to a lock-in amplifier as a detector (Figure 2). A pulsed Nd: YAG laser (Q-smart 850 mJ, Quantel Laser) with its harmonics (213, 266, 355, and 532 nm) is used for the multiwavelength excitation. The microphone (Knowles FG 23742 D36), with a homemade power supply of 3 V and a lock-in amplifier (MFLI 500 kHz, Zurich Instruments) operating in the kHz frequency range, has been used as the photoacoustic detector. UV-grade fused silica windows and lenses (L 1 and L 2) are used for all optical components. Further details about the PA cell and setup are given elsewhere.^17^

### Sample Information

Breath samples are collected and stored in inert 2L Tedlar bags (Techinstro Industries, Nagpur) with an integrated valve. Exhaled breath from three deep exhales is collected in the Tedlar bag, and the measurements are made within an hour. To avoid dietary influences on the breath VOCs; subjects were advised to avoid any food consumption for a minimum of 4 h before sample collection.^22^

To check the performance of breath analysis with different laser excitations, samples from the same subjects have been tested (five each from normal and disease (common cold)). Asthma and COPD samples were recorded with the assembled PAS setup with only 266 nm laser excitation, as explained later. Exhaled breath samples were collected from the Department of Respiratory Medicine at Kasturba Hospital, Manipal. Ethical approval for breath sample collection was obtained from the Institutional Ethics Committee (IEC 1000/2019) and the Indian Council of Medical Research (ICMR) (CTRI/2020/02/031357), the Government of India. Each volunteer received a participant information sheet containing study details, and informed consent was obtained from the volunteers before collecting samples.

We collected breath samples from volunteers representing health conditions such as asthma (24), COPD (20), and normal lung function (25), resulting in a comprehensive cohort of 69 subjects. For the thorough evaluation and comparison of our experimental technique, each sample underwent both PAS and E-nose analyses, and measurements were repeated 5 times, respectively. Asthma and COPD patients were identified through the F_E_NO test, which revealed concentrations of NO in their breath samples exceeding 40 ppb. The normal volunteers participating in the study were determined to be clinically healthy. Detailed participant information is available in Table S1.

The breath samples collected in Tedlar bags were introduced into the setup. Subsequently, a 10 s sample purge was performed, during which the PA signal was recorded. This was followed by a 60 s ambient air purge to clean the PAS cell. The experiments were conducted within a laboratory environment set at a temperature of 20 °C and a humidity level of 56%. Throughout the experiments, the gas pressure was consistently maintained at atmospheric pressure equivalent to that of the exhaled breath samples. A commercial E-nose (Cyranose-320) is the device used to confirm and compare the results obtained from PAS. Cyranose-320 is suitable for classifying lung cancer, COPD, asthma, and post-COVID syndrome.^6,23,24^

### Data Processing

**Figure 3:**
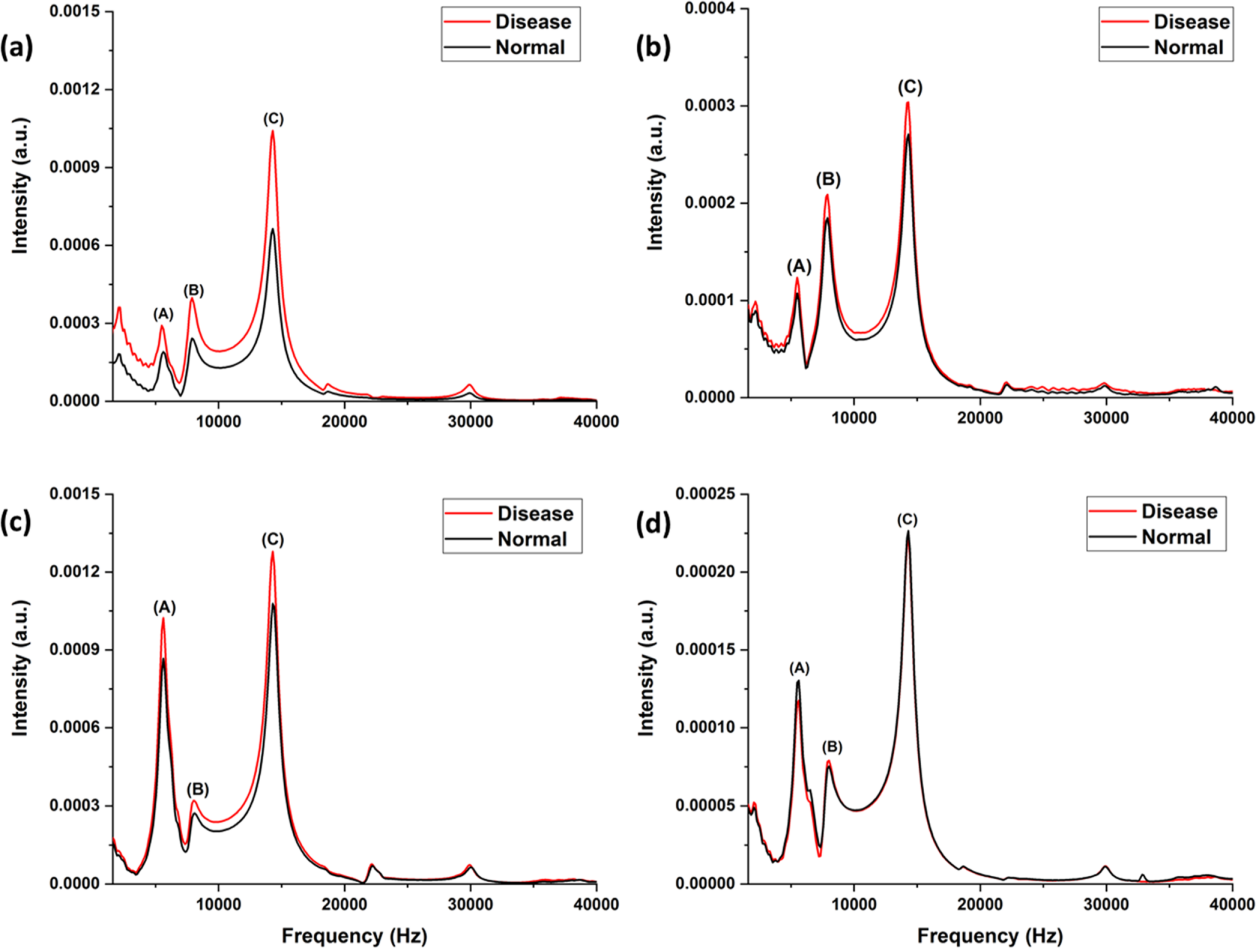
Average PAS signal of disease (common cold) and normal samples using (a) 213, (b) 266 nm, (c) 355, and (d) 532 nm laser excitations.

To highlight the distinctions among the different sample categories, the averages of the PA signals are presented in Figure 3. The peaks A, B, and C are the acoustic modes of the PA cell. Descriptive statistics was performed to understand the difference between normal and disease samples for different laser excitations.^25^ Descriptive statistics have been performed for the highest peak of the PAS data (peak C) for different laser excitations. The purpose of descriptive statistics is to provide a concise summary and analysis of a data set. It helps in understanding the central tendency (mean, median, and mode), variability (range, variance, and standard deviation), and data distribution.

Principal component analysis (PCA) is a reliable method for simplifying complex data sets by creating new, independent variables while preserving important information. By changing the original data into these new variables (principal components (PC1, PC2, PC3···..)) that are at right angles to each other, PCA makes it easier to understand, visualize, and model the data, which helps to uncover hidden patterns and relationships that can be hard to see in data with many dimensions.^26^ PAS data from breath samples has been normalized by using peak A. Later, the data was interpolated within the frequency range of 1600–40000 Hz, and PCA was performed using Origin software. A plot of PC1 against PC2 was generated to visualize the classification. A similar analysis was carried out for the PAS data recorded using 266 nm laser excitation involving samples from volunteers with asthma, COPD, and normal lung function.

In the context of disease marker exploration, current practices often limit measurement to one or two markers due to the time and resource constraints of conventional methods. However, clinical samples such as exhaled breath consist of a multitude of markers, some disease-specific, others shared among diseases, and some unrelated to the ailment. A “Match/No-match” test approach is proposed to address this complexity. This involves creating meticulously curated “Standard Calibration” sample groups with confirmed normal and disease samples. By subjecting these samples to PCA, statistical metrics like “Mahalanobis distance” (M. distance) and “spectral residuals” are derived. Testing samples are then compared against this standard set using PCA.^27^ The test sample’s M. distance and spectral residuals are compared to the standard metrics within a set deviation range. This method optimizes all PCA-contributing factors and utilizes adjustable statistical parameters, offering autonomy in AI/ML-driven decision-making free from observer dependency.

The Match/No-match test was performed on PAS data utilizing Grams AI software.^6,17,25^ A calibration set was prepared by using 90 normal photoacoustic data. Subsequently, for the Match/No-match test, a total of 255 data were collected from normal (35), asthma (120), and COPD (100) conditions and subjected to analysis. Similarly, distinct calibration sets were prepared using asthma and COPD data and then independently tested, as illustrated in Table 1. Rejection values for M distance have been established for each model, considering the data distribution. From the results of the Match/No-match test, sensitivity and specificity are calculated for each calibration set. The process for computing sensitivity and specificity for the normal calibration set is outlined as follows:
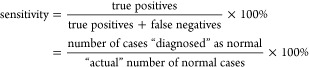

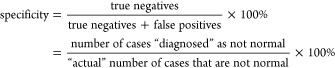
Utilizing sensitivity and specificity derived from the Match/No-match analysis and M. distance results, the receiver operating characteristics (ROC) curve has been plotted for each model.^28−30^ This ROC curve aids in assessing the method’s performance for analysis. The area under the curve of the ROC (AUC-ROC) is computed to quantify this performance. The AUC-ROC value ranges from 0 to 1, with higher values signifying the superior diagnostic capability of the model for the samples in question.

**Table 1:** Match/No-Match Test Results

calibration set (total no., calibn. set no.)	class	match (count)	M. distance range (rejection value)	S. residual range	sensitivity %	specificity %
normal (125, 90)	asthma	yes (0)	1.43–7.50	2.18 × 10^–09^–1.76 × 10^–08^	88.57	89.09
		no (120)				
	COPD	Yes (24)	1.21–3.63	1.34 × 10^–09^–8.01 × 10^–09^		
		no (76)				
	normal	yes (31)	4.10 × 10^–02^–1.04 (1.2)	–1.15 × 10^–09^–3.65 × 10^–09^		
		no (4)				
asthma (120, 85)	asthma	yes (30)	0.12–1.30 (1.4)	–5.12 × 10^–10^1.92 × 10^–09^	85.71	84
		no (5)				
	COPD	yes (36)	1.40–8.36	–3.81 × 10^–09^–1.97 × 10^–08^		
		no (64)				
	normal	yes (0)	1.47–21.50	3.78 × 10^–09^–4.70 × 10^–08^		
		no (125)				
COPD (70, 30)	asthma	yes (20)	1.00–8.15	1.33 × 10^–09^–1.99 × 10^–08^	86.66	85.71
		no (100)				
	COPD	yes (26)	0.22–0.94 (1.0)	1.01 × 10^–09^–3.67 × 10^–09^		
		no (4)				
	normal	yes (15)	1.03–3.65	1.08 × 10^–09^–1.00 × 10^–08^		
		no (110)				

## Results and Discussion

### Excitation Wavelength Optimization

**Figure 4:**
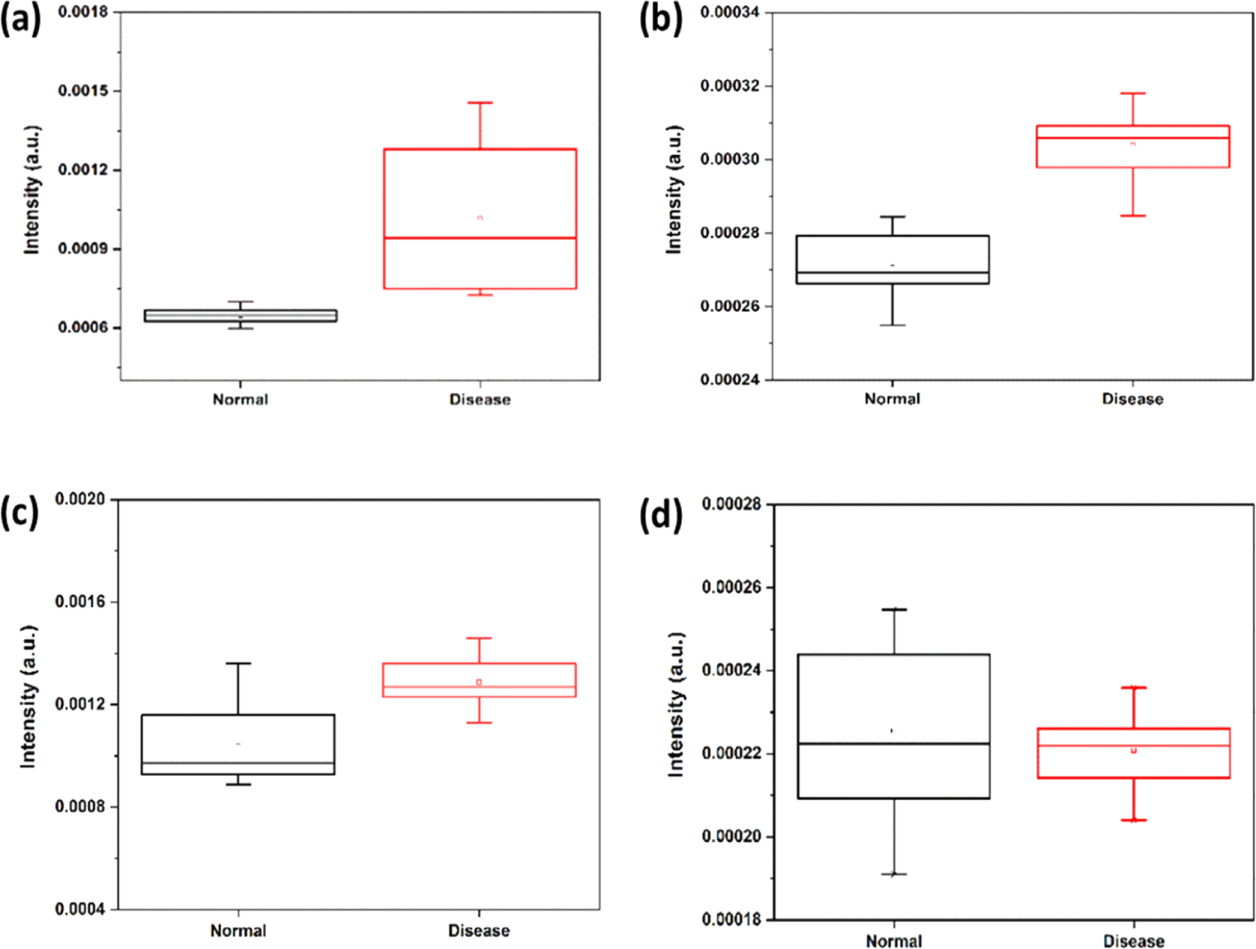
Descriptive statistics for the peak C of PAS signal of disease (common cold) and normal samples using (a) 213, (b) 266 nm, (c) 355, and (d) 532 nm laser excitations.

Averaged PA signals of disease (common cold) and normal samples and corresponding descriptive statistics are shown in Figures 3 and 4, respectively, for different laser excitations. In this study, we considered the mean, major distribution of the data, and maximum and minimum from the data sets. From descriptive statistics, it is observed that 213, 266, and 355 nm laser excitations show considerable differences in the PA signal of normal and disease samples, the signal from samples in disease condition being always larger than the signal from normal sample, indicating the presence of compounds which absorb more of the radiation at these excitation wavelengths. The PAS signal (Figure 3(d)) with 532 nm excitation has shown a negligible difference between disease and normal samples. This is due to VOCs’ absence of absorption at 532 nm in the exhaled breath samples. The signal in this case, much smaller than that observed at other excitation wavelengths, is mostly from effects like window-heating, scattered radiation, and components in ultratrace quantities, the signal from which overlapped with the larger signals of other components and was overridden by the much stronger signals from the sample at other excitation wavelengths.

A closer examination of the signal at 532 nm excitation shows that the peak at around 5500 Hz is stronger for normal samples, showing the opposite effect compared to the results at other wavelengths, supporting such a possibility.

**Figure 5:**
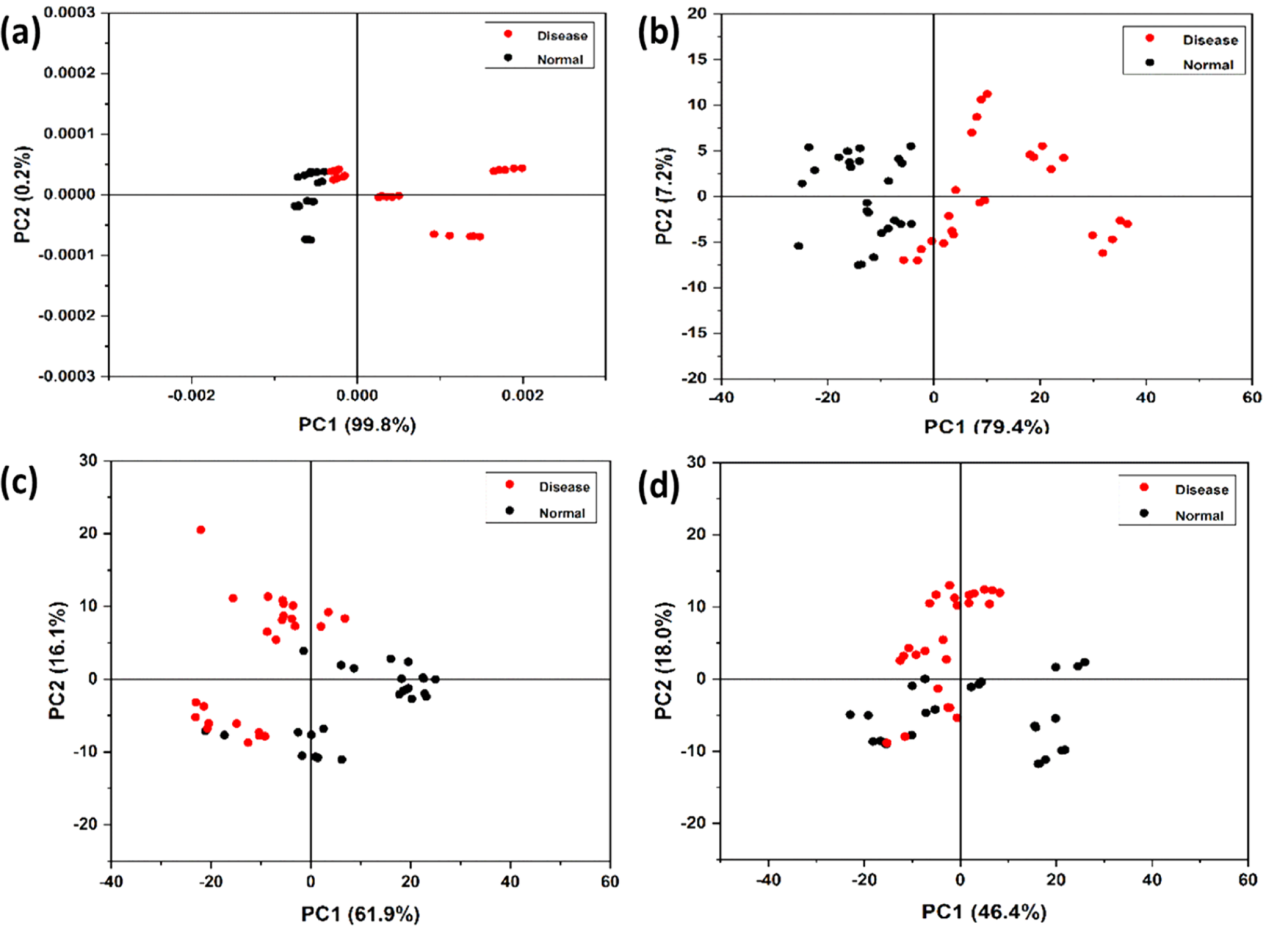
PCA of PAS signal of disease (common cold) and normal samples with (a) 213, (b) 266 nm, (c) 355, and (d) 532 nm laser excitations.

PCA was carried out for the “disease”, and normal samples were recorded using 213, and 266 nm. 355 and 532 nm laser excitations are shown in Figure 5a–d, respectively. It has been observed that UV lasers at 213, 266, and 355 nm are more suitable for PA measurements, indicating that many of the VOCs absorb in that region. Comparatively, fewer variations were observed for disease and normal samples at 532 nm laser excitation. NO is a common marker associated with any airway disorders/respiratory issues,^31^ it has strong absorption around 213 and 355 nm and comparatively weak absorption at 266 nm.^32^ Here the classification observed for 266 nm (Figure 4b) is from the absorption of the VOCs present in the breath sample. It was also observed that many standard VOCs (e.g., acetone) have shown good PA signal for 266 nm compared to other wavelengths, which is evident from the absorption spectra of standard VOC samples (Figure S1). In view of the above, further studies with asthma and COPD were performed with 266 nm excitation only.

### PAS Study of Asthma, COPD, and Normal Samples with 266 nm Excitation

**Figure 6:**
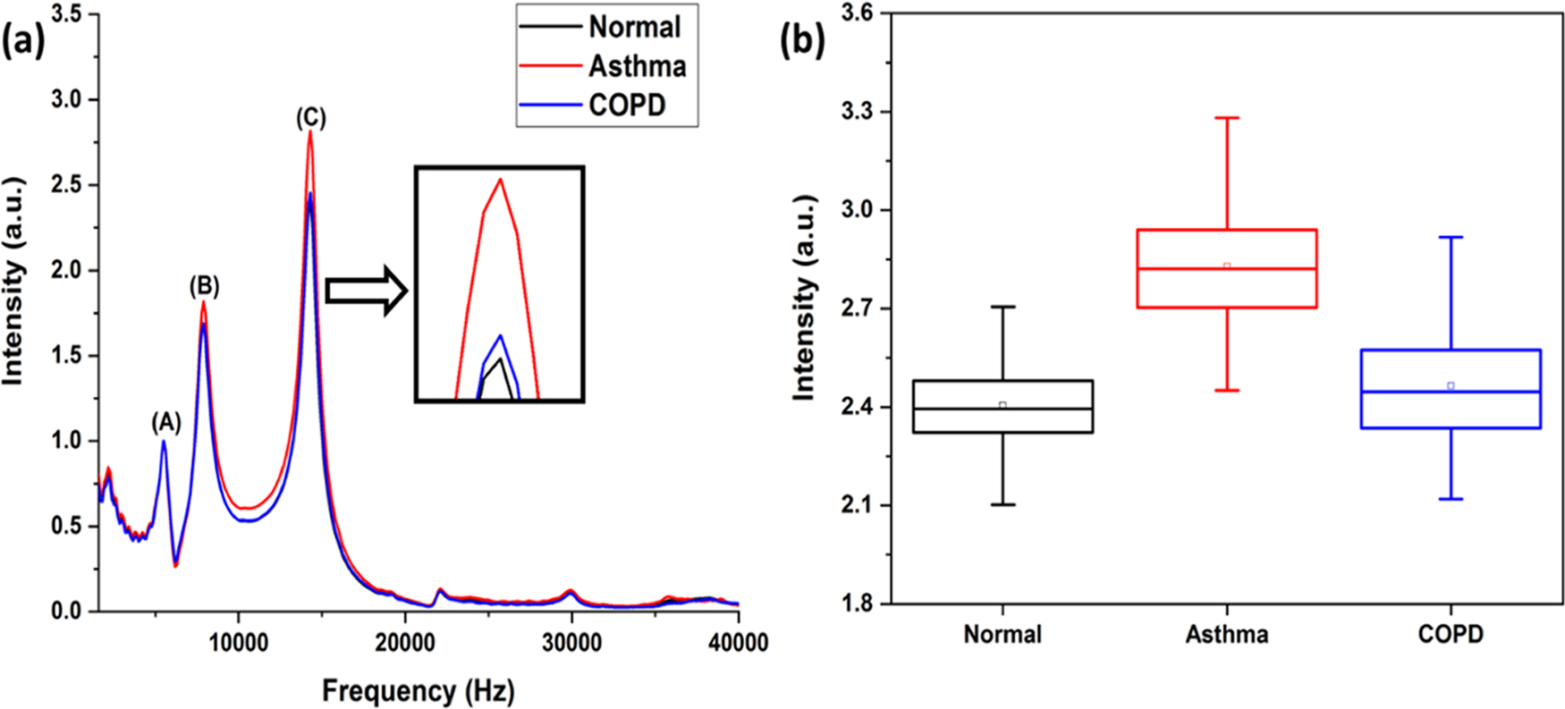
(a) Average PA signal with 266 nm excitation of asthma, normal, and COPD samples (normal and COPD are almost overlapped), where A, B, and C are the acoustic modes. (b) Descriptive statistics of the acoustics mode C.

Asthma, COPD, and normal samples were excited with 266 nm excitation, and the “Mean” PA signals for them are shown in Figure 6(a). To understand the difference in relative intensities, descriptive statistics of asthma, COPD, and normal samples are performed for the acoustics mode C, which is shown in Figure 6(b). It is seen that the relative intensity variations observed for the three classes of samples show varying results arising from the absorption-relaxation characteristics of the different VOCs present in the three different sets of samples. However, the normal and COPD samples show very similar grouping, though both differ noticeably from the asthma group. The PAS data have been further analyzed using PCA and the Match/No-match test, to check how far the PAS results are suitable for diagnosing different health conditions.

**Figure 7:**
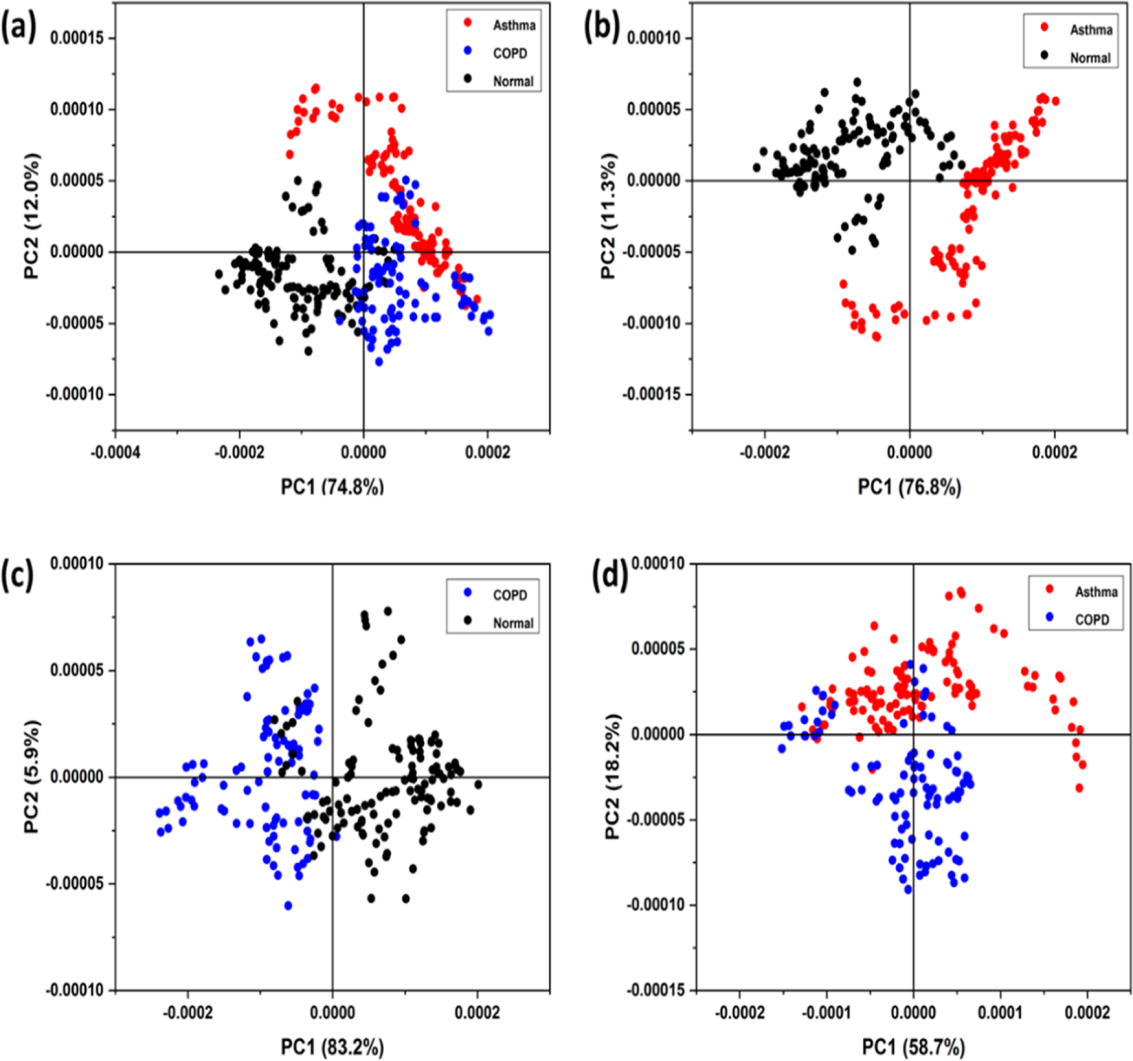
Score plot (PC1 vs PC2) obtained from principal component analysis of (a) asthma, COPD, and normal, (b) asthma and normal, (c) COPD and normal, and (d) asthma and COPD.

PCA was performed for asthma, normal, and COPD data; the results are shown in Figure 7. As expected, PC1 is the major factor in the classifications of the different classes of samples. It is also seen that although there is some overlap between the asthma and COPD samples, they are reasonably well-separated (Figure 7(d)), which is important for the objective diagnosis of diseases.

**Figure 8:**
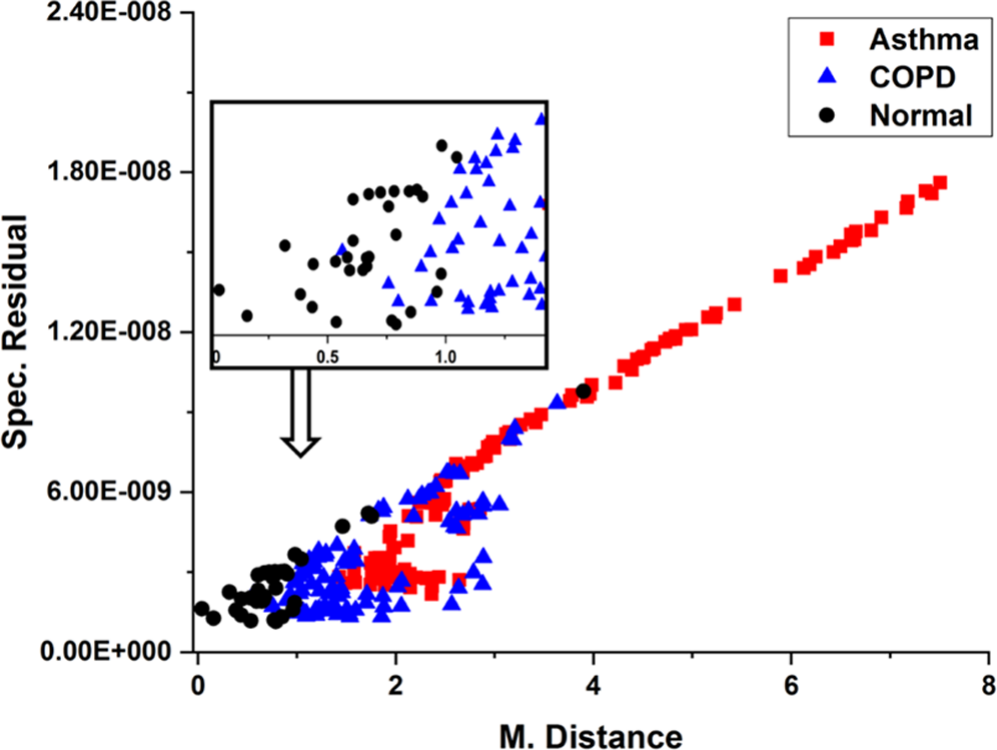
M. distance vs spectral residual plot from the Match/No-match results for the normal calibration set.

Match/No-match studies were performed using calibration sets from each of the asthma, COPD, and normal data. Test summaries, sensitivity, and specificity have been calculated from the Match/No-match outcome and are shown in Table 1. All the calibration sets can be used for diagnostic applications, in which a normal calibration set has better sensitivity and specificity. Figure 8 displays a plot representing spectral residual and M. distance values, as obtained from the Match/No-match test involving samples from individuals with asthma, COPD, and normal respiratory conditions using a normal calibration set.

**Figure 9:**
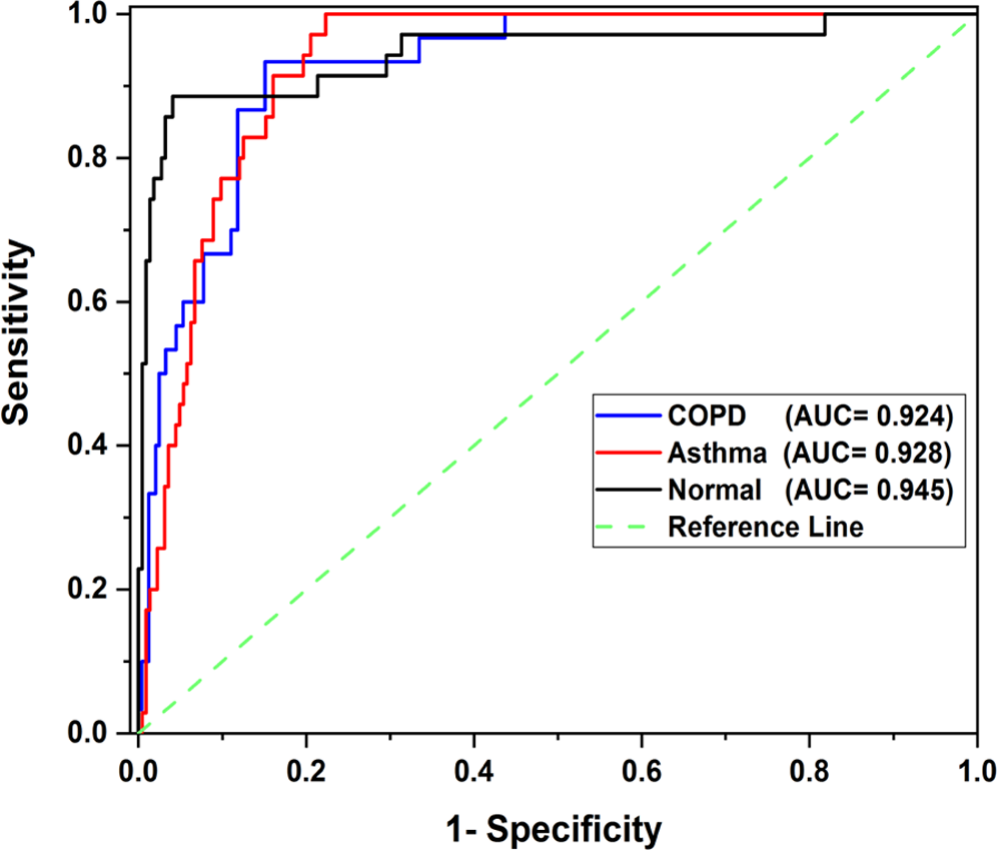
ROC curve obtained with the M. Distance values.

ROC curves for normal, asthma, and COPD calibration sets are plotted in Figure 9. It is observed that the AUC-ROC values, corresponding to normal, asthma and COPD calibration sets, are 0.948, 0.928, and 0.924, respectively. These ROC values indicate a high confidence level in diagnosing asthma and COPD using the PAS breath analysis technique.

### Breath Analysis Using E-Nose

**Figure 10:**
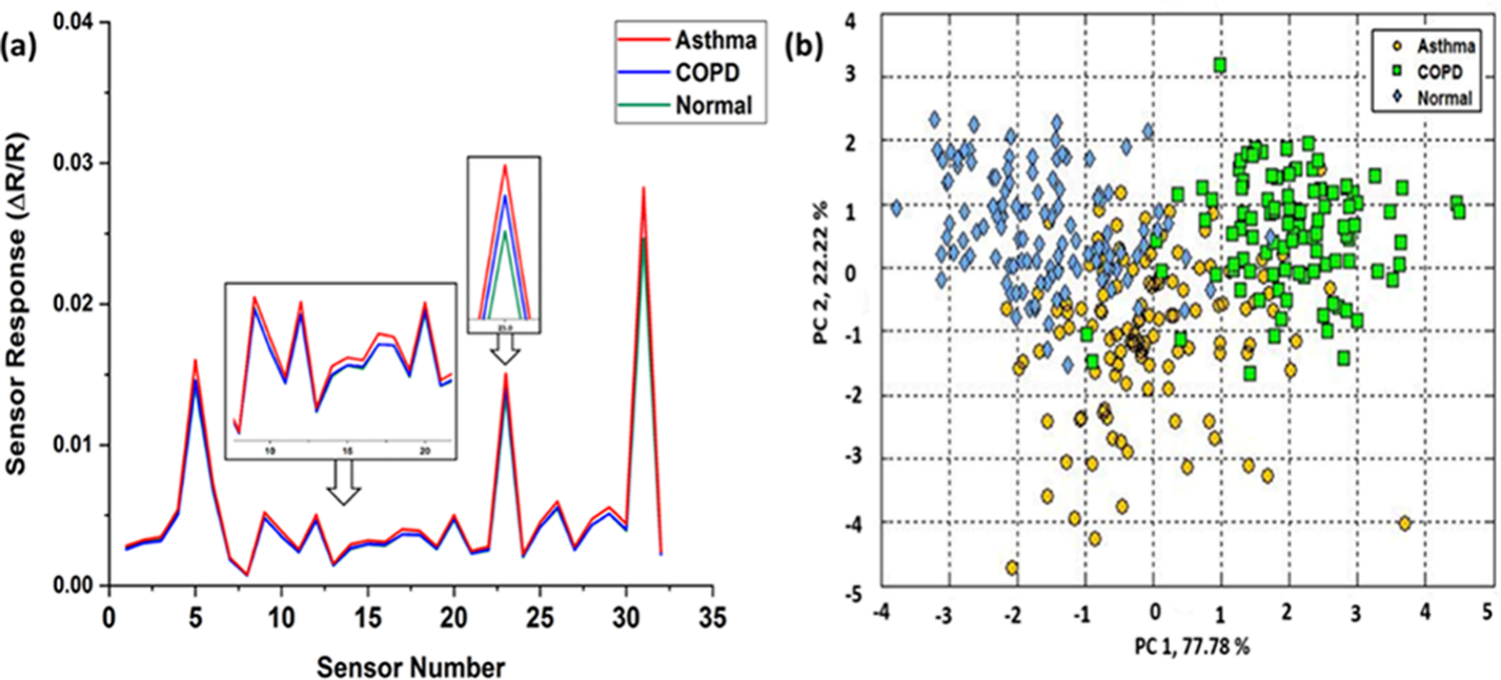
(a) Sensor response of asthma, COPD, and normal breath samples, (b) PC 1 vs PC 2 plot of the PCA of the E-nose data performed for asthma, COPD, and normal.

We also compared the PA analysis results of the exhaled breath samples with results from a commercial E-nose device. Earlier studies using this E-nose device demonstrated its ability to diagnose asthma conditions.^24,33,34^ The same breath samples from COPD, asthma, and normal subjects used for the PAS study were tested by using E-nose. The samples were recorded five times each and Figure 10 (a) shows the average sensor response of asthma, COPD, and normal breath samples. It is observed that similar to the PA signal, the asthma samples have an increased signal intensity compared to the other two, and the COPD and Normal samples give signals very close to each other. PCA study of the data was carried out using CD analysis software. Again, as observed in the PAS - PCA study, discrimination among the samples is supported by the E-nose-based experiment (Figure 10(b)) with some overlap among the COPD and asthma samples, similar to that observed in PAS. The summary of the Match/No-match study performed for the E-nose data from asthma, COPD, and normal samples is shown in Table S2. Sensitivities of 85.71, 86.66, and 83.33 were obtained for normal, asthma, and COPD calibration sets, respectively. The PAS device developed for asthma and COPD diagnosis has better sensitivity and specificity than the E-nose device in view of the highly selective UV laser absorption of VOCs with 266 nm excitation.

It is seen that though there is a reasonable amount of discrimination among the three classes, there is also a noticeable overlap between the asthma and other groups. A possible reason could be that, since the E-noses are devices manufactured with specific elements intended for “application specific” use, one requires different sets of sensors for different applications, whereas PAS is a spectroscopic technique, applicable universally, the spectra providing the specificity, in each case. Even though E-nose devices can serve as convenient tools for universal screening, UV-PAS with single laser excitation can be used for screening applications where multiple conditions corresponding to different diseases have to be discriminated from one another in the same observation (e.g., in various lung disorders like asthma, COPD, PCS, various cancers, etc.). The photoacoustic spectroscopy method utilized here is very sensitive and highly suitable because the absorption maxima of several VOC markers are around 266 nm, which may be one of the reasons for the discrimination of diseases.

## Summary

Breath analysis has immense potential to diagnose asthma and COPD. At present, F_E_NO is the routine method used for diagnosing asthma and COPD. Due to the presence of numerous other VOC biomarkers in exhaled breath, relying solely on F_E_NO for a comprehensive assessment of the disease, including disease stages and associated metabolic changes, can be challenging. Though methods like GC-MS involve costly and bulky setups and are suitable mainly for lab-based applications, but not for situations like universal screening, PoC applications, etc. Similarly, selective, low detection sensitivity (in ppm only) and short shelf life make E-noses unsuitable for routine applications. The PAS method is highly objective, can give observer-independent results by AI/ML methods, and with recent advances in spectroscopic instrumentation make it cost-effective, portable, and allows PoC application.

In this work, studies to identify the suitable wavelengths for absorption studies in the UV–Vis spectral range on breath samples have been carried out, since UV–Vis spectroscopy has several advantages like much higher sensitivity due to the much larger absorption cross sections compared to IR, possibility of multimodal (absorption, fluorescence), as well as multiwavelength operation. Using the multiwavelength technique, in the present studies it is found that the UV laser excitations give better results for breath samples. The 266 nm laser excitation gives good classification due to the absorption of VOCs (other than NO) present in human breath. The data fusion method can also be attempted for better classification by combining data with different laser excitations, and exploring UV LEDs as excitation sources for miniaturization is the future scope of the study.

Asthma and COPD are the most common respiratory problems that always need attention in diagnosis since the situation can deteriorate and become life-threatening. Clinical breath samples from asthma, COPD, and normal subjects were collected and recorded with the assembled PAS setup. Match/No-match study performed for asthma, COPD, and normal data gives good discrimination of one class from the other with sensitivity and specificity values of 88% and 89% using a normal calibration set. AUC of ROC values (>0.9 for asthma and COPD) shows the diagnostic capability of the system for asthma and COPD. All the results obtained from the PAS sensor are comparable, even better than that with the E-nose.

## Supporting Information Available

The following files are available free of charge. The Supporting Information is available free of charge at https://pubs.acs.org/doi/10.1021/acssensors.3c01316.

Figure S1. Normalized UV–Vis absorption spectra in the range (190–600 nm) of standard VOC samples; Table S1. Participant details of asthma, COPD, and normal subjects for the PAS study; Table S2. Summary of the Match/No-match study for E-nose data (PDF)

## References

[ref1] Adigal S S.; V R N.; John R. V.; Pai K. M.; Bhandari S.; Mohapatra A. K.; Lukose J.; Patil A.; Bankapur A.; Chidangil S. A review on human body fluids for the diagnosis of viral infections: scope for rapid detection of COVID-19. Expert Rev. Mol. Diagn. 2021, 21 (1), 31–42. 10.1080/14737159.2021.1874355. 33523770

[ref2] Nidheesh V. R.; Mohapatra A. K.; VK U.; Sinha R. K.; Nayak R.; Kartha V. B.; Chidangil S. Breath analysis for the screening and diagnosis of diseases. Appl. Spectrosc. Rev. 2021, 56 (8–10), 702–732. 10.1080/05704928.2020.1848857.

[ref3] John R. V.; Devasiya T.; Nidheesh V. R.; Adigal S.; Lukose J.; Kartha V. B.; Chidangil S. Cardiovascular biomarkers in body fluids: progress and prospects in optical sensors. Biophys. Rev. 2022, 14 (4), 1023–1050. 10.1007/s12551-022-00990-2. 35996626 PMC9386656

[ref4] Sukul P.; Schubert J. K.; Zanaty K.; Trefz P.; Sinha A.; Kamysek S.; Miekisch W. Exhaled breath compositions under varying respiratory rhythms reflects ventilatory variations: translating breathomics towards respiratory medicine. Sci. Rep. 2020, 10 (1), 14109 10.1038/s41598-020-70993-0. 32839494 PMC7445240

[ref5] Das S.; Pal M. Non-invasive monitoring of human health by exhaled breath analysis: A comprehensive review. J. Electrochem. Soc. 2020, 167 (3), 037562 10.1149/1945-7111/ab67a6.

[ref6] Nidheesh V. R.; Mohapatra A. K.; VK U.; Lukose J.; Kartha V. B.; Chidangil S. Post-COVID syndrome screening through breath analysis using electronic nose technology. Anal. Bioanal. Chem. 2022, 414 (12), 3617–3624. 10.1007/s00216-022-03990-z. 35303135 PMC8930465

[ref7] Risby T. H.; Solga S. F. Current status of clinical breath analysis. Appl. Phys. B: Laser Opt. 2006, 85, 421–426. 10.1007/s00340-006-2280-4.

[ref8] Guntner A. T.; Abegg S.; Konigstein K.; Gerber P. A.; Schmidt-Trucksass A.; Pratsinis S. E. Breath sensors for health monitoring. ACS Sens. 2019, 4 (2), 268–280. 10.1021/acssensors.8b00937. 30623644

[ref9] Rothbart N.; Stanley V.; Koczulla R.; Jarosch I.; Holz O.; Schmalz K.; Hübers H. W. Millimeter-wave gas spectroscopy for breath analysis of COPD patients in comparison to GC-MS. J. Breath Res. 2022, 16 (4), 046001 10.1088/1752-7163/ac77aa. 35688126

[ref10] Rothbart N.; Holz O.; Koczulla R.; Schmalz K.; Hübers H. W. Analysis of human breath by millimeter-wave/terahertz spectroscopy. Sensors 2019, 19 (12), 2719 10.3390/s19122719. 31212999 PMC6630364

[ref11] Kartha V. B.; Santhosh C. Biomedical Spectroscopy; Manipal University Press, 2014.

[ref12] Li B.; Wu H.; Feng C.; Jia S.; Dong L. Noninvasive Skin Respiration (CO2) Measurement Based on Quartz-Enhanced Photoacoustic Spectroscopy. Anal. Chem. 2023, 95 (14), 6138–6144. 10.1021/acs.analchem.3c00536. 36987565

[ref13] Popa C.; Patachia M.; Banita S.; Matei C.; Bratu A. M.; Dumitras D. C. The level of ethylene biomarker in the renal failure of elderly patients analyzed by photoacoustic spectroscopy. Laser Phys. 2013, 23 (12), 125701 10.1088/1054-660X/23/12/125701.

[ref14] Cristescu S. M.; Kiss R.; te Lintel Hekkert S.; Dalby M.; Harren F. J.; Risby T. H.; Marczin N. Real-time monitoring of endogenous lipid peroxidation by exhaled ethylene in patients undergoing cardiac surgery. Am. J. Physiol.: Lung Cell. Mol. Physiol. 2014, 307 (7), L509–L515. 10.1152/ajplung.00168.2014. 25128523 PMC4187041

[ref15] Neerincx A. H.; Mandon J.; van Ingen J.; Arslanov D. D.; Mouton J. W.; Harren F. J.; Merkus P. J.; Cristescu S. M. Real-time monitoring of hydrogen cyanide (HCN) and ammonia (NH3) emitted by Pseudomonas aeruginosa. J. Breath Res. 2015, 9 (2), 027102 10.1088/1752-7155/9/2/027102. 25634638

[ref16] Narasimhan L. R.; Goodman W.; Patel C. K. Correlation of Breath Ammonia with Blood Urea Nitrogen and Creatinine During Hemodialysis, Apr 10, Proceedings of the National Academy of Sciences, 2001; pp 4617–4621. 10.1073/pnas.071057598 PMC31883 11296293

[ref17] Nidheesh V. R.; Mohapatra A. K.; Nayak R.; Unnikrishnan V. K.; Kartha V. B.; Chidangil S. UV laser-based photoacoustic breath analysis for the diagnosis of respiratory diseases: Detection of Asthma. Sens. Actuators, B 2022, 370, 132367 10.1016/j.snb.2022.132367.

[ref18] WH Organisation. Asthma. 2023 (accessed May 15, 2023).

[ref19] Ratiu I. A.; Ligor T.; Bocos-Bintintan V.; Mayhew C. A.; Buszewski B. Volatile organic compounds in exhaled breath as fingerprints of lung cancer, asthma and COPD. J. Clin. Med. 2021, 10 (1), 32 10.3390/jcm10010032. PMC7796324 33374433

[ref20] Christiansen A.; Davidsen J. R.; Titlestad I.; Vestbo J.; Baumbach J. A systematic review of breath analysis and detection of volatile organic compounds in COPD. J. Breath Res. 2016, 10 (3), 034002 10.1088/1752-7155/10/3/034002. 27578038

[ref21] Vogelmeier C. F.; Roman-Rodriguez M.; Singh D.; Han M. K.; Rodriguez-Roisin R.; Ferguson G. T. Goals of COPD treatment: focus on symptoms and exacerbations. Respir. Med. 2020, 166, 105938 10.1016/j.rmed.2020.105938. 32250871

[ref22] Belluomo I.; Boshier P. R.; Myridakis A.; Vadhwana B.; Markar S. R.; Spanel P.; Hanna G. B. Selected ion flow tube mass spectrometry for targeted analysis of volatile organic compounds in human breath. Nat. Protoc. 2021, 16 (7), 3419–3438. 10.1038/s41596-021-00542-0. 34089020

[ref23] Dragonieri S.; Annema J. T.; Schot R.; van der Schee M. P.; Spanevello A.; Carratú P.; Resta O.; Rabe K. F.; Sterk P. J. An electronic nose in the discrimination of patients with non-small cell lung cancer and COPD. Lung Cancer 2009, 64 (2), 166–170. 10.1016/j.lungcan.2008.08.008. 18834643

[ref24] Plaza V.; Crespo A.; Giner J.; Merino J. L.; Ramos-Barbón D.; Mateus E. F.; Torrego A.; Cosio B. G.; Agustí A.; Sibila O. Inflammatory Asthma Phenotype Discrimination Using an Electronic Nose Breath Analyzer. J. Invest. Allergol. Clin. Immunol. 2015, 25 (6), 431–437. 26817140

[ref25] Nidheesh V. R.; Mohapatra A. K.; Nayak R.; Unnikrishnan V. K.; Kartha V. B.; Chidangil S. Bimodal UV photoacoustic and fluorescence sensor for breath analysis. Sens. Actuators, B 2023, 379, 133242 10.1016/j.snb.2022.133242.

[ref26] Greenacre M.; Groenen P. J.; Hastie T.; d’Enza A. I.; Markos A.; Tuzhilina E. Principal component analysis. Nat. Rev. Methods Primers 2022, 2 (1), 100.

[ref27] Adigal S S.; Bhandary S. V.; Hegde N.; Nidheesh V. R.; John R. V.; Rizvi A.; George S. D.; Kartha V. B.; Chidangil S. Protein profile analysis of tear fluid with hyphenated HPLC-UV LED-induced fluorescence detection for the diagnosis of dry eye syndrome. RSC Adv. 2023, 13 (32), 22559–22568. 10.1039/D3RA04389D. 37501778 PMC10369224

[ref28] Zou K. H.; O’Malley A. J.; Mauri L. Receiver-operating characteristic analysis for evaluating diagnostic tests and predictive models. Circulation 2007, 115 (5), 654–657. 10.1161/CIRCULATIONAHA.105.594929. 17283280

[ref29] Bradley A. P. The use of the area under the ROC curve in the evaluation of machine learning algorithms. Pattern Recognit. 1997, 30 (7), 1145–1159. 10.1016/S0031-3203(96)00142-2.

[ref30] Metz C. E. Basic principles of ROC analysis. Semin. Nucl. Med. 1978, 8, 283–298. 10.1016/s0001-2998(78)80014-2. 112681

[ref31] Proud D. Nitric oxide and the common cold. Curr. Opin. Allergy Clin. Immunol. 2005, 5 (1), 37–42. 10.1097/00130832-200502000-00008. 15643342

[ref32] Lagesson-Andrasko L.; Lagesson V.; Andrasko J. The Use of Gas-Phase UV Spectra in the 168– 330-nm Wavelength Region for Analytical Purposes. 1. Qualitative Measurements. Anal. Chem. 1998, 70 (5), 819–826. 10.1021/ac971009v. 21644613

[ref33] Dragonieri S.; Quaranta V. N.; Carratu P.; Ranieri T.; Resta O. Exhaled breath profiling by electronic nose enabled discrimination of allergic rhinitis and extrinsic asthma. Biomarkers 2019, 24 (1), 70–75. 10.1080/1354750X.2018.1508307. 30074408

[ref34] Tenero L.; Sandri M.; Piazza M.; Paiola G.; Zaffanello M.; Piacentini G. Electronic nose in discrimination of children with uncontrolled asthma. J. Breath Res. 2020, 14 (4), 046003 10.1088/1752-7163/ab9ab0. 32512553

