# Supplementary material for: Multiwavelength Photoacoustic Breath Analysis Sensor for the Diagnosis of Lung Diseases: COPD and Asthma
Source: ACS Sens. 2023 Oct 23;8(11):4111–20. doi: 10.1021/acssensors.3c01316 (PMC10683506; doi:10.1021/acssensors.3c01316)
Supplement: Supplementary file 1 — se3c01316_si_001.pdf [file se3c01316_si_001.pdf]

Supporting Information

Multi-wavelength photoacoustic breath analysis sensor for the diagnosis of lung diseases: COPD and asthma

Nidheesh V. R.[1], Aswini Kumar Mohapatra[2], Vasudevan Baskaran Kartha[1], and Santhosh Chidangil[1*]

1. Centre of Excellence for Biophotonics, Department of Atomic and Molecular Physics, Manipal Academy of Higher Education, Manipal, Karnataka, India-576104

2. Department of Respiratory Medicine, Kasturba Medical College, Manipal, Manipal Academy of Higher Education, Manipal, Karnataka, India-576104

*Corresponding author: santhosh.cls@manipal.edu

Figure S1: Normalized UV-Vis absorption spectra in the range (190 nm – 600 nm) of standard VOC samples.

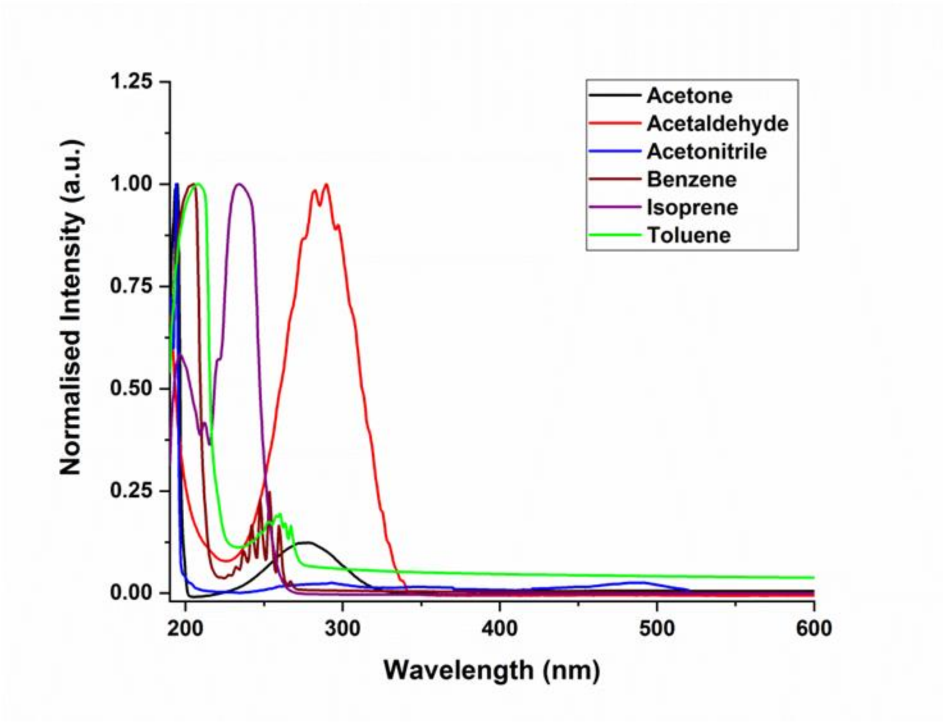

Table S1 : Participant details of asthma, COPD and normal subjects for PAS study.

Sample Number	Habits/History (smoking, tobacco chewing, alcohol,)	Sex (Male - M) (Female - F)	Age	Clinical diagnosis
1	None	F	52	Normal
2	None	F	40	Normal
3	None	F	27	Normal
4	None	M	28	Normal
5	None	M	26	Normal
6	None	F	29	Normal
7	None	F	23	Normal
8	None	M	33	Normal
9	None	M	28	Normal
10	None	M	28	Normal
11	None	F	24	Normal
12	None	M	26	Normal
13	None	M	28	Normal
14	None	F	28	Normal
15	None	F	29	Normal
16	None	M	35	Normal
17	None	M	34	Normal
18	None	F	40	Normal
19	None	F	24	Normal
20	None	M	34	Normal
21	None	M	32	Normal
22	None	F	50	Normal
23	None	F	48	Normal
24	None	M	34	Normal
25	None	M	40	Normal
26	None	M	40	Asthma
27	None	F	61	Asthma
28	Tobacco chewing	F	60	Asthma
29	None	F	60	Asthma
30	None	M	64	Asthma
31	None	F	58	Asthma
32	None	F	54	Asthma
33	None	F	31	Asthma
34	Tobacco chewing	M	60	Asthma
35	Alcohol	M	38	Asthma
36	None	M	67	Asthma
37	None	F	36	Asthma
38	None	M	87	Asthma
39	None	M	64	Asthma

40	None	M	72	Asthma
41	None	M	70	Asthma
42	None	F	37	Asthma
43	None	M	72	Asthma
44	None	F	23	Asthma
45	None	F	71	Asthma
46	None	F	44	Asthma
47	None	F	37	Asthma
48	Tobacco chewing	M	38	Asthma
49	None	M	37	Asthma
50	None	M	78	COPD
51	Tobacco chewing	M	64	COPD
52	None	M	72	COPD
53	Smoking	M	68	COPD
54	None	F	57	COPD
55	None	M	75	COPD
56	None	M	74	COPD
57	None	M	70	COPD
58	Tobacco chewing	M	79	COPD
59	None	M	78	COPD
60	Smoking	M	63	COPD
61	None	M	65	COPD
62	Tobacco chewing	M	77	COPD
63	Tobacco chewing	M	73	COPD
64	None	M	72	COPD
65	None	M	62	COPD
66	Smoking	M	62	COPD
67	Tobacco chewing	F	71	COPD
68	None	F	67	COPD
69	None	F	62	COPD

Table S2: Summary of the Match/No-match study for E-nose data.

Calibration set	Class	Match (count)	M.distance range (Rejction value)	Spec. Residual range	Sensitivity %	Specificity %
Normal	Asthma	Yes (13)<br>No (107)	0.520346-<br>10.93457	1.42E-07-<br>3.26E-05	85.71	87.27
	COPD	Yes (15)<br>No (85)	0.501113-<br>9.147009	1.40E-07-<br>2.72E-05		
	Normal	Yes (30)<br>No (5)	3.70E-02-<br>0.48831 (0.5)	4.53E-07 -<br>3.35E-06		
Asthma	Asthma	Yes (26)<br>No (4)	8.47E-02-<br>0.498902 (0.5)	8.68E-07 -<br>3.48E-06	86.66	78.22
	COPD	Yes (24)<br>No (76)	0.507612-<br>3.971861	2.11E-07 -<br>2.10E-05		
	Normal	Yes (25)<br>No (100)	0.503688-<br>2.775883	2.98E-07-<br>1.52E-05		
COPD	Asthma	Yes (18)<br>No (102)	0.4019- - 7.7588	1.27E-06-<br>3.25E-05	83.33	87.75
	COPD	Yes (25)<br>No (5)	0.11229-<br>0.37548 (0.4)	1.02E-07-<br>1.42E-06		
	Normal	Yes (12)<br>No (113)	0.41865-<br>4.66508	1.73E-06-<br>2.05E-05		
